# Targeting of the anti-apoptotic gene survivin in human thyroid carcinoma

**DOI:** 10.3892/ijmm.2012.1046

**Published:** 2012-06-28

**Authors:** ZHOUXUN CHEN, NAXIN LIU, GUANBAO ZHU, HENNING DRALLE, CUONG HOANG-VU

**Affiliations:** 1 Department of General Surgery, The First Affiliated Hospital of Wenzhou Medical School, Wenzhou, Zhejiang 325000, P.R. China;; 2 Research Group of Experimental and Surgical Oncology, Department of General, Visceral and Vascular Surgery, Martin Luther University Halle-Wittenberg, D-06097 Halle, Germany

**Keywords:** inhibitor of apoptosis, survivin, thyroid carcinoma, prognostic factor, FTC-133 cell line, small interfering RNA

## Abstract

Survivin is a novel apoptosis inhibitor. Its gene is related to the baculovirus gene, which is believed to play a crucial role in fetal development and in cancer. We attempted to determine the expression of survivin in both thyroid goiter and carcinoma tissues, and to evaluate its prognostic value in human thyroid disease. In the present study, we applied small interfering RNA (siRNA) directed against survivin to determine the effects of decreasing the high constitutive levels of this protein in the FTC-133 thyroid follicular cancer cell line. Using reverse transcription PCR and immunohistochemistry, we compared the expression of survivin with relevant clinical and pathological data of 90 postsurgical specimens from patients with primary thyroid carcinoma and patients with benign goiter (33 with papillary thyroid cancer, 24 with follicular thyroid cancer, 18 with undifferentiated thyroid cancer and 15 cases with goiter). For the siRNA treatment in a human follicular thyroid carcinoma cell line, fluorescein-labeled double-stranded ultrapure siRNAs were used. RT-PCR identified the survivin transcript in 67/75 (89.3%) tumor samples and in 4/15 benign goiter samples. Immunohistochemical analysis showed positive immunoreactivity in 65/75 (86.7%) carcinomas while no expression was noted in all of the 15 benign goiter tissues. Survivin mRNA and protein levels were significantly higher in cancer tissues compared to benign goiter tissues (P<0.001). Higher survivin expression was found in the tumor tissues of pT3/pT4 and in the tumors with lymph node metastasis (P<0.05). Tumors with distant metastasis demonstrated higher survivin expression compared to the tumors without distant metastasis. Additionally, the expression of survivin in undifferentiated carcinomas was higher than that in differentiated ones. There was no significant correlation between survivin expression and age, gender, histological subtype and pathological stage. Our additional studies demonstrated that siRNA directed against survivin markedly decreased the protein expression of survivin. In conclusion, we conclude that survivin expression indicates more aggressive behavior and metastatic ability in thyroid cancer cells *in vivo*. Survivin can be used as a diagnostic and therapeutic marker for thyroid carcinoma and an important target in the strategy of thyroid cancer therapy. Our results of siRNA silencing indicate that siRNA may have potential as a therapeutic modality in the treatment of human thyroid cancer.

## Introduction

Cell death is an essential phenomenon for cell homeostasis, as well as for cell growth. It has been well documented during embryonic and postembryonic development (1,2). The control of cell division by programmed cell death (apoptosis) is indispensable for the preservation of the normal growth and specialization of an organism. Several proteins that inhibit apoptosis, such as p53 and Bcl-2 family, are involved in the regulation of normal cellular homeostasis and the promotion of tissue tumorgenesis (3–5). The physiological apoptotic pathways are often altered in malignant cells, resulting in a significant advantage in survival. Tumor cells that are resistant to apoptosis can survive despite immune system tumor surveillance, and often fail to respond to anticancer treatment.

Human survivin is a cytoplasmic protein with a molecular weight of 16.5 kDa. As a member of the inhibitor of apoptosis protein (IAP) family, it has been identified in baculovirus (6). It consists of 142 amino acids, and its gene spans 14.7 kb on the telomeric position on chromosome 17, to band q25 (7). The human survivin gene consists of 4 exons and 3 introns, and its coding strand contains an open reading frame (ORF) of 426 nucleotides complementary to EPR-1 (7). There is a TATA-less promoter and there are GC-rich regions of canonical CpG islands 25 upstream of this ORF (7–9). Survivin has recently been identified as a novel IAP. Unlike other members of the IAP family, survivin is characterized by a unique structure that contains a single baculovirus IAP repeat and no really interesting new gene (RING) finger motifs (10).

Survivin is expressed during embryonal development and in many common types of human cancers including stomach (11), colorectal (12), lung (13), breast (14), pancreatic (15) and prostate cancers (16) and high-grade non-Hodgkin’s lymphomas *in vivo* (7,17). Nevertheless, its expression is absent in terminally differentiated adult tissues (7). It is expressed fetally but not in adult differentiated tissues. Recent results suggest that survivin may counteract a default induction of apoptosis in the G2/M phase (18) in proliferating cells. Survivin is expressed in a cell cycle-regulated manner with high levels in G2/M and rapid downregulation following cell cycle arrest. At the beginning of mitosis, survivin associates with the mitotic spindle and disruption of this interaction results in a loss of its anti-apoptotic function. Overexpression of survivin in cancer may thus overcome this apoptosis-related cell cycle checkpoint and favor aberrant progression of transformed cells through mitosis.

Two major apoptosis pathways, the mitochondrial pathway and the death receptor pathway, are known. Bcl-2 which blocks release of mitochondrial cytochrome c into the cytosol has been shown to inhibit the first of these two pathways (19,20). The apoptosis pathway is blocked by survivin. Survivin inhibits apoptosis by directly inhibiting downstream effectors caspase-3 and -7 through baculovirus IAP repeat (BIR)-dependent recognition (14), and by interfering with caspase-9 activity processing. Survivin blocks a common downstream part of both the mitochondrial apoptosis pathway and the death receptor pathway (21,22).

The RNA interference (RNAi) phenomenon is a recently observed process in which the introduction of a double-stranded RNA (dsRNA) into a cell causes the specific degradation of an mRNA with the same sequence. The 21–23 nt guide RNAs, small RNAs generated by RNase III cleavage from longer dsRNAs, are associated with sequence-specific mRNA degradation. Small RNAs have been proposed as gene expression repressors with great potential for use in gene therapy (10,23,24). This technique known as RNA interference has been successfully adapted to mammalian cells so that it is now possible to decrease the expression of cellular genes specifically after transfection of annealed small interfering 21-mer RNAs. In the present study, we aimed to ascertain whether we could specifically reduce the levels of the survivin protein in follicular thyroid cancer cell line FTC-133, which overexpresses survivin protein. For this analysis, RNAi using designed small interfering RNAs (siRNAs) directed against survivin was carried out (25,26).

We performed this retrospective study of thyroid carcinoma patients for the purpose of investigating whether survivin expression is signficantly associated with poor prognosis and whether it may be used as a potential therapeutic target for thyroid tumors.

## Materials and methods

### Tissue specimens

Tissue specimens from 75 patients with thyroid carcinoma (33 with papillary thyroid cancer (PTC), 24 with follicular thyroid cancer (FTC), 18 with undifferentiated thyroid cancer (UTC) and 15 patients with benign thyroid goiter, 35 males and 52 females, were studied. This study was approved by the local committee of medical ethics and all patients provided written consent. Tissues of all patients were obtained following surgery performed between 2001 and 2006 at the Department of General, Visceral and Vascular Surgery, Martin Luther University Halle-Wittenberg, Halle/Saale, Germany. The mean age of the patients was 58 years, with a range of 15–89 years. For immunohistochemistry and RT-PCR, resected thyroid tissues were immediately frozen in liquid nitrogen and maintained at −80°C until they were used. Frozen sections (6 μm) were cut on a cryostat, and control sections were stained with hematoxylin and eosin (H&E).

### mRNA preparation and RT-PCR analysis

Total-RNA from fresh thyroid carcinoma, benign goiter tissues and human colorectal carcinoma cell line ‘Caco’ (as positive control) was extracted using the TRIzol reagent (Invitrogen, Carlsbad, CA, USA) according to the manufacturer’s protocol. Reverse transcription was performed from 1 μg of total-RNA by using the Superscript II kit (Gibco, Munich, Germany) at 42°C for 30 min, followed by enzyme inactivation at 95°C for 5 min.

For PCR amplification, a 2 μl aliquot of the reaction mixture was used. To obtain reproducible quantitative performance of the multiplex RT-PCR assay, we titrated the amount of starting cDNA and adjusted the number of amplification cycles. The generated cDNA was amplified using the specific primer for survivin and for the housekeeping gene β-actin. The primers used in this study and the expected size from the reported cDNA sequence are shown in Table I.

All subsequent assays were carried out under conditions that amplification of both survivin and β-actin (the internal control) was yielded within a parallel linear range. The PCR profile was as follows: 30 sec at 94°C, 30 sec at 60°C, 45 sec at 72°C, 7 min at 72°C and a final step at 6°C. All PCR reactions were carried out with AmpliTaq Gold (Amersham, USA). After 38 PCR amplification cycles, 20 μl of PCR-amplified cDNA had migrated on a 1% agarose gel and bands were visualized with ethidium bromide, photographed with Kodak Image System 440 cf and electronically evaluated with Kodak Digital Science 1D software (Eastman Kodak, New Heaven, CT, USA).

The human colorectal carcinoma cell line ‘Caco’ exhibited strong survivin-mRNA expression. Its expression level was set as 100%. The expression levels of all investigated specimens were classified in comparison to the positive control grey scale as follows: 0–20%, negative (−); 20–50%, decreased (+), 50–75% moderate expression (++), 75%, strongly positive (+++).

### Immunohistochemistry

To confirm the results of survivin gene expression obtained by RT-PCR, immunohistochemistry was performed using Dako coverplates (Dako, Germany). Cryo-embedded serial sections (6 μm) of all tissues were freshly cut and then incubated in a mixture of 3% H_2_O_2_ and ice cold 90% methanol (1:4) for 20 min. After twice washing with PBS solution, sections were incubated in PBS solution for 10 min. Enzymatic activity and non-specific binding sites were blocked with normal swine serum (1:4 diluted) in 1% PBS-BSA for 10 min to suppress non-specific binding. Subsequently, replicate sections were incubated at 4°C overnight with the specific mouse monoclonal antibody against human survivin (clone D8; Santa Cruz Biotechnology, Inc., Santa Cruz, CA, USA) at a dilution of 1:200. Negative control sections were exposed to the secondary antibody only and processed as described above. After the sections were washed 3 times in PBS, they were incubated for 30 min at a 1:1,000 dilution of biotinylated goat anti-mouse secondary antibody (Dako anti-IgG kit). Detection of antibody-antibody-antigene reaction was accomplished using the avidin-biotin-peroxidase complex method. A 15% solution of diaminobenzidine (DAB) (Dako, Aarhus, Denmark) was used as chromogen. Finally, sections were lightly counterstained with Mayer’s hematoxylin. Tissue sections from a human colorectal adenocarcinoma were used as positive controls.

### Evaluation of immunostained tissues

All sections were examined by two independent reviewers. For better quantification, planimetric measurement of immunoreactive cell clusters and tissue parts was evaluated semi-quantitatively, using an Axioplan light microscope (Zeiss, Jena, Germany) by three independent investigators, blinded to the histological diagnosis. In addition, planimetric evaluations on immunostained specimens were performed using Zeiss KS300 software, and the plasma-nucleus relationship of survivin-positive cells was documented. For better quantification, planimetric measurement of immunoreactive cell clusters and tissue parts was performed using PalmRobo-Software (Palm Microlaser Technologies, Tutzing, Germany). The numbers of survivin-positive cells were calculated after circumferential allignment in relation to microscope magnification. Amount of positive squares was set in contrast to the total section area (TSA=100%), and the level of staining intensity was subdivided into four groups: 0, 0–10% negative; 1, >10–50% weak; 2, >50–80% distinct; and 3, >80% strong.

### Cell lines

The human follicular thyroid carcinoma cell line FTC-133, supplied by P. Goretzki (Düsseldorf, Germany), was established from a 42-year-old male patient with metastatic FTC, characterized by expressing human thyroglobulin and thyroid peroxidase. The cell line was cultured in Dulbecco’s modified Eagle’s medium (DMEM) and modified HAM-F12 medium 1:1 supplemented with 10% fetal bovine serum and penicillin/streptomycin in a humidified incubator at 37°C in 5% CO_2_. Media were changed every 3–4 days.

### Preparation of siRNAs

siRNAs with two thymidine residues (dTdT) at the 3-end of the sequence were designed using the designing siRNA program of Qiagen for survivin (5′-AAGGACCACCGCATCTCTACA-3′) (Qiagen-Xeragon, USA), which extends between 92–112 nucleotides of the coding mRNA sequence of survivin (NCBI accession no. NM001168.1).

Double-stranded ultrapure siRNAs (HPLC-purified >97% pure) were generated by mixing the corresponding pair of sense and antisense RNA oligonucleotides. These were fluorescein-labeled as RNAs, dissolved in 1 ml of the provided sterile buffer (100 mM potassium acetate, 30 mM HEPES-KOH, 2 mM magnesium acetate, pH 7.4) to give a 20 μM solution. The reaction mixture was heated to 90°C for 1 min, allowed to incubate at 37°C for 60 min, and then aliquoted and stored at −20°C. A non-specific (mismarch) siRNA (Qiagen-Xeragon) served as the negative control.

### Treatment of cells with fluorescence-labeled siRNAs

One day prior to transfection, approximate 4×10^5^ FTC-133 cells were plated per 6-well plate in 2 ml DMEM media containing 10% fetal bovine serum and antibiotics to achieve 40–60% confluence. Cells were incubated under normal growth conditions (generally 37°C and 5% CO_2_). Transfection of the siRNAs was performed using TransMessenger™ transfection reagent (Qiagen, Hilden, Germany). Two types of siRNA concentrations (135 pM, code no. sis-100 and 270 pM, code no. sis-248) were used in this study. According to the manipulation, siRNAs were incubated with Enhancer R, TransMessenger transfection reagent and cell growth medium without serum and antibodies. The cells were then washed once with pre-warmed (37°C) PBS. After washing, 1 ml transfection mix was added to each well. The cells were incubated for 4 h at 37°C, in 5% CO_2_. Following the incubation, the cells were washed once again with pre-warmed (37°C) PBS. Subsequently, the transfection medium was replaced by normal growth medium DMEM containing 10% FCS and antibodies.

Survivin mRNA and protein levels were compared in the untreated and mock-transfected cells using RT-PCR and immunocytochemistry at 24 and 72 h and 7 days post-transfection. The results were confirmed in three independent experiments.

### Measurement of the intensity of survivin staining by immunocytochemistry

For RT-PCR and the immunofluorescence assay, the cells were harvested at different time points 24 and 72 h and 1 week post-transfection. Total-RNA from fresh cells was extracted using the TRIzol reagent (Invitrogen) according to the manufacturer’s protocol.

To confirm the effect of siRNA directed against survivin, immunocytochemistry was performed using Dako coverplates (Dako). At 24 and 72 h and 7 days after siRNA-transfection, cells were immediately frozen in liquid frozen medium (42.8 g saccharose + 0.33 g MgCl_2_ in 250 ml PBS + 250 ml glycerol) and maintained at −20°C until use. The manipulation followed the same steps as the immunohistochemistry assay.

### Statistical analysis

All experimental and clinical-pathological parameters were calculated for statistical significance using SigmaPlot 8.0 software (SPSS Inc., Chicago, IL, USA), and the 2-tailed unpaired t-test was used to compare the statistical significance of the differences in data from two groups, where appropriate. P-values of <0.05 were considered to indicate statistical significance.

## Results

### mRNA expression in thyroid carcinoma and benign goiter tissues analyzed by RT-PCR

**Figure 1.**
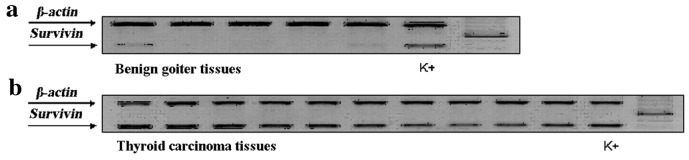
Survivin mRNA expression in thyroid tissues. (a) Weak or negative expression of survivin mRNA in benign goiter tissues; (b) strong survivin mRNA expression in carcinoma tissues. K^+^, positive control, human colorectal carcinoma cell line ‘Caco’.

**Figure 2.**
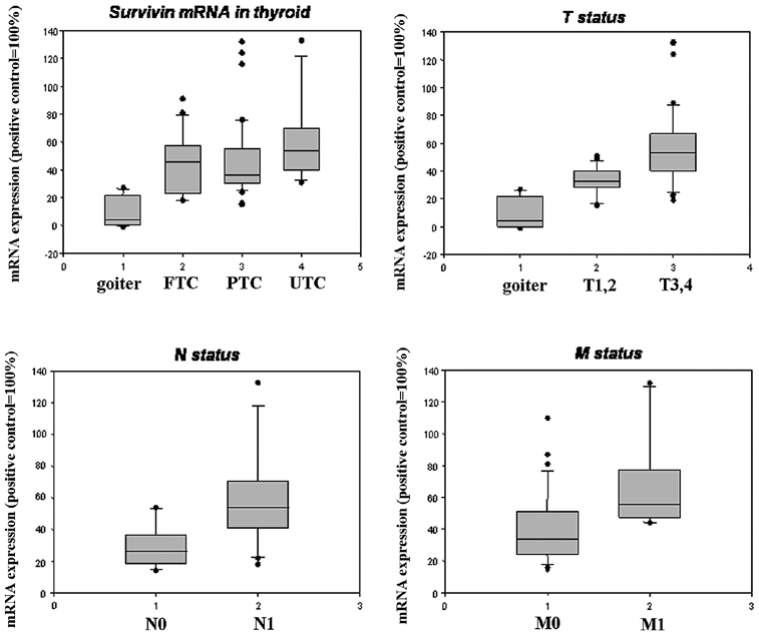
Correlation between the transcript expression of endogenous survivin and various clinicopathological parameters. Thyroid cancer samples expressed higher transcript expression of endogenous survivin when compared to the benign goiters; the expression was significantly correlated with the grade of tumor invasiveness (pT), local lymph node metastasis (N) and distant metastasis (M) of the thyroid carcinoma tissues.

Transcripts of survivin in all of the goiter tissues were evaluated as weak or negative, in contrast, moderate or strong expression of survivin was observed in the carcinoma tissues (Fig. 1). In general, RT-PCR identified the survivin transcript in 67/75 (89.3%) of the tumors. However, extremely weak expression of survivin mRNA was noted in 4/15 of the benign goiter samples. Expression of survivin mRNA was significantly higher in the thyroid carcinoma tissues than that in the benign goiter tissues. Moderate or strong positive survivin expression was noted in 24/57 (42.1%) differentiated carcinomas and in 12/18 (66.7%) undifferentiated carcinomas. The expression level of survivin in undifferentiated carcinomas was generally higher than that in differentiated carcinomas (P>0.05). The relationship between survivin mRNA expression and various prognostic factors are documented in Table II and Fig. 2. A correlation was found between various pT stages (pT1/2-pT3/4, P=0.009), the presence of lymph node metastases (N0-N1, P=0.035) and distant metastasis (M0-M1, P=0.08) in the thyroid carcinoma cases, while no significant correlation was found in relation to age, gender and pathological subtype.

### Protein expression in thyroid carcinoma and benign goiter tissues analyzed by immunohistochemistry

**Figure 3.**
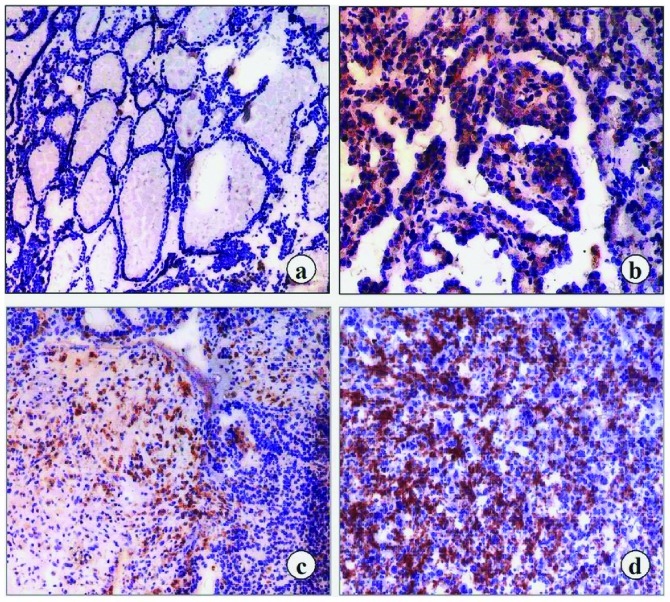
Immunostaining patterns of survivin in frozen human thyroid tissue samples. (a) Thyroid goiter tissue with a loss of expression of survivin. (b) Strong immunostaining in a primary PTC (pT4N0M0). (c) A primary FTC (pT4N1M0) showing moderate survivin protein expression. (d) Uniformly intense staining of survivin protein expression in UTC (pT4N1M0) tissue. PTC, papillary thyroid cancer; FTC, follicular thyroid cancer; UTC, undifferentiated thyroid cancer.

**Figure 4.**
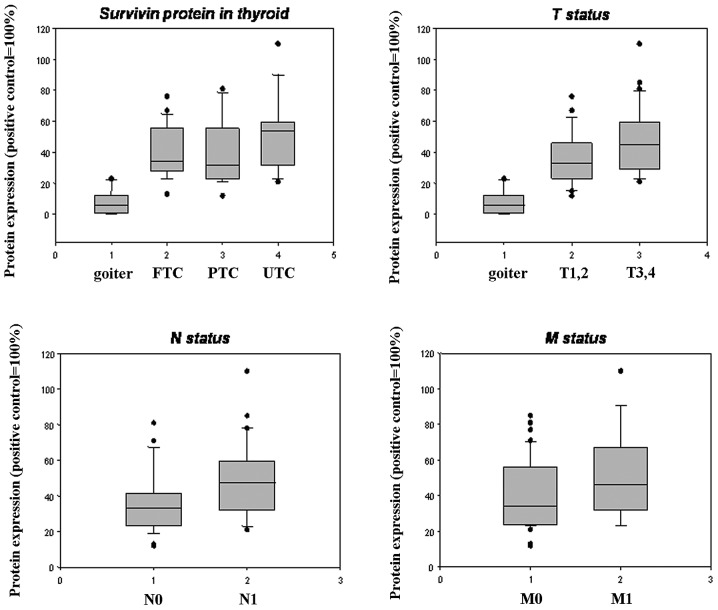
Correlation between the protein expression of endogenous survivin and various clinicopathological parameters. Protein expression of endogenous survivin was significantly correlated with the grade of tumor invasiveness T and local lymph node metastasis (N); and with distant metastasis (M) of thyroid carcinoma tissues but this value did not achieve statistical significance.

Examples of survivin immunostaining are shown in Fig. 3. The results of the immunohistochemical study demonstrated good correlation with that of the RT-PCR analysis. Among the tumors examined, 65/75 (86.7%) carcinomas revealed survivin immunoreactivity in the cytoplasm of the tumor cells, whereas no expession was found in all of the 15 normal and benign goiter tissues. A uniformly intense survivin protein expression was detected in the cellular cytoplasm of the differentiated thyroid carcinoma tissues, and the staining often appeared granular. A similar but stronger pattern was observed in the UTC tissues. Following comparison of the different pathological features of the carcinoma cases, the UTC cases exhibited the strongest positive survivin immunoreactivity while the PTC and FTC cases displayed high or moderate levels of survivin. Among the 40 examined primary tumors without metastasis, 17 cases (∼42.5%) showed weak or moderate expression. In 25/35 (∼71.4%) primary tumors with regional lymph node infiltration and distant metastasis, moderate or high survivin immunostaining was noted. We found a similar correlation with mRNA expression between the different pT stages (pT1/2–pT3/4, P<0.001), N status (N0–N1, P=0,011), M status (M0–M1, P<0.05) in differentiated and undifferentiated thyroid carcinoma. However, there was no statistically significant difference between survivin expression and the other clinicopathologic features. The relationship between survivin protein expression and various prognostic factors are shown in Table II and Fig. 4. Therefore, dedifferentiation of thyroid carcinoma cells may cause an increase in the expression of survivin transcripts and immunoreactive protein.

### Downregulating effects of siRNA on survivin

**Figure 5.**
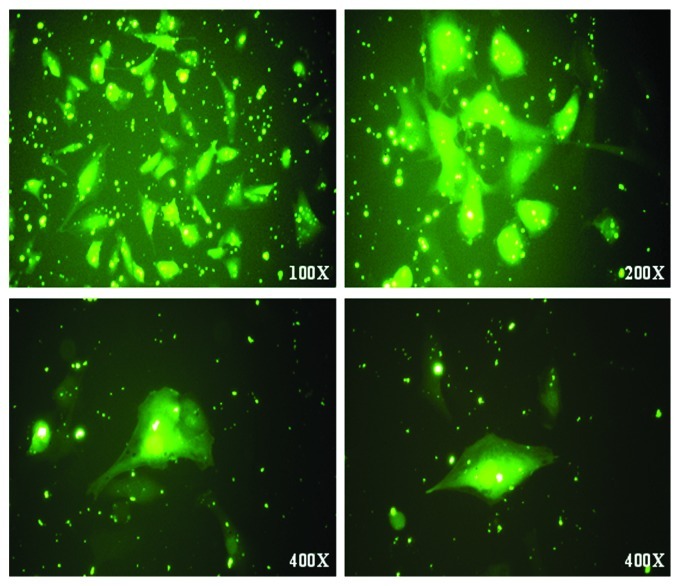
Transfection efficiency of siRNA of FTC-133 cells. Approximately 60% of whole cells were transfected with fluorescein-labeled siRNA (upper left panel, x100 magnification; upper right panel, x200 magnification). A ginkgo biloba formed single transfected cell and a spindle-shaped cell are shown in the lower panels (x400 magnification).

Fluorescein signal was detected by fluorescence microscopy as a granular pattern in the cytoplasm surrounding nuclei (Fig. 5). Green fluorescein-positive cells and total cells were counted in 10 randomly selected fields. The transfection efficiency was calculated to be ∼45–55%.

**Figure 6.**
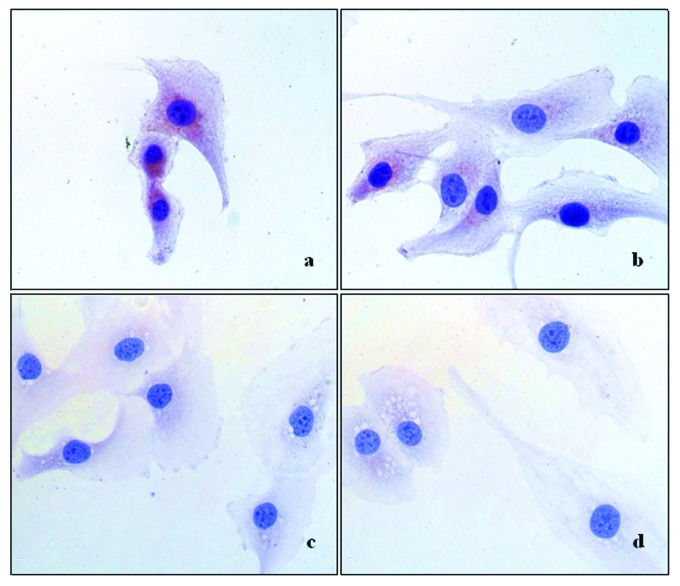
Effect of survivin siRNA on FTC-133 cells as detected using immunocytochemistry. (a) Untreated cells; (b) cells treated with mismatch siRNA; (c) cells treated with sis-100 siRNA; (d) cells treated with sis-248 siRNA

The effects of survivin siRNA treatment on the cells were visualized by immunocytochemistry. Survivin immunostaining was undetectable in the cells which were treated with survivin siRNA (Fig. 6c and d) on day 1, 3 and 7 following transfection. In contrast, all cells from untreated and control experiments retained strong staining (Fig. 6a and b).

**Figure 7.**
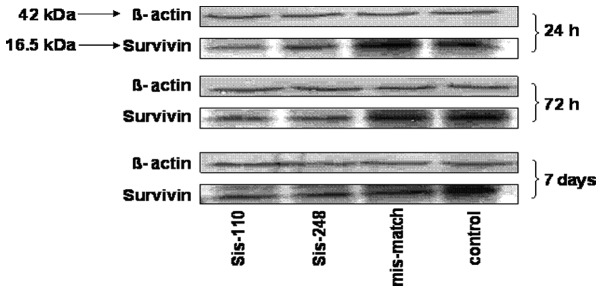
Western blot analysis indicates reduced survivin expression following siRNA treatment in FTC-133 cells.

For a quantitative evaluation of endogenous survivin protein expression in the FTC-133 cells, immunoblotting (western blot analysis) was performed according to the protocol described above. Total proteins in the cells were extracted from a 6-well cell plate after 24 and 72 h and 1 week of transfection. Twenty-four hours after transfection, a maximum downregulation to 46% of the initial protein level was achieved at 24 h in sis-110 treated cells (∼50% in comparison with untreated cells) and sis-248 (∼70%) (Fig. 7). Decreased survivin levels were still observed after 72 h. Additionally, this silencing was still noted 7 days following siRNA treatment (60%). Different siRNAs had no influence on endogenous β-actin expression. Its expression was equal in each experimental sample.

## Discussion

Several studies have shown a prominent correlation between survivin expression and tumor aggressiveness (7,11–14,16). Thus, we postulated prior to this study that the level of survivin expression may be a significant indicator for the progression of thyroid carcinoma. The results of the present study suggest that decreased apoptosis integrated partly by survivin expression is a predictive indicator of poorer prognosis in patients with thyroid carcinoma.

In the present study, we extended initial observations and clearly demonstrated that survivin mRNA and protein were expressed consistently and highly in thyroid carcinoma tissues, while all goiter specimens exhibited the absence or significant downregulation of survivin expression and were considered to be survivin-negative, with the exception of weak expression in four tissue specimens. At present, it is unclear why several normal and benign cells exhibited survivin expression. A possible explanation is that the mRNA sample containing survivin mRNA may have been obtained from mitotically active cells, as survivin has been shown to regulate the cell cycle in the G2-M phase. The presence of an invasive phenotype such as lymph node infiltration or distant metastasis coincided with strong expression of survivin. By contrast, cases exhibiting weak expression of survivin were devoid of metastases (N0/M0), suggesting a role for survivin in the tissue invasiveness of thyroid carcinoma. We found no correlation between the expression of survivin in regards to age and gender. However, the expression of survivin appears to be correlated with tumor size, regional lymph node metastasis, distant metastasis and different pathological subtypes.

To our knowledge, the present study represents the first investigation of the expression of survivin in thyroid carcinoma. In conclusion, the present results suggest that survivin is upregulated during thyroid carcinoma progression. Our data demonstrated that survivin has an increased expression profile in advanced stages of thyroid carcinoma such as pT3/T4. Furthermore, the diminished expression of survivin is associated with metastasis and increased expression of survivin may represent a potentially useful prognostic marker for the classification, staging and subtyping of human thyroid carcinomas. Elucidation of the mechanisms of survivin and prediction of whether its expression proves useful for the clinical treatment of thyroid cancer patients warrants further investigation.

The possible role of survivin as a target for cancer vaccines in different types of cancers has been discussed (25,27–29). Therefore, in the present study, the siRNA transfection experiment using a thyroid carcinoma cell line was performed in our laboratory. The RNAi effect was demonstrated by transfecting siRNA into FTC-133 cells where it significantly reduced expression of the survivin gene. Expression was not suppressed in cells that were untransfected, or transfected with a nonspecific control siRNA. We confirmed the downregulation of survivin expression by use of siRNA to block survivin mRNA and protein expression. Notably, siRNA survivin does not induce death in a normal cell population due to the absence of survivin expression in normal cells. Our data suggest that the use of siRNA survivin warrants further investigation as a novel approach to selective cancer therapy.

## Figures and Tables

**Table I.** PCR primers and conditions.

Gene	Primer	Temperature (°C)	Product size (bp)
Survivin	5′-AAC AGC CGA GAT GAC CTC C-3′		
	5′-AAC TTC AGG TGG ATG AGG AGA C-3′	60	398
β-actin	5′-GCT GGA AGT GGA CAG CGA-3′		
	5′-GGC ATC GTG ATG GAC TCC G-3′	60	608

**Table II.** Relationship of survivin expression and various prognostic factors in 75 patients with thyroid carcinoma.

Clinico-pathological characteristics	No. of patients (total)	Survivin mRNA positive (++, +++)	Survivin mRNA decreased (−, +)	P-value	Survivin protein positive (2, 3)	Survivin protein decreased (0, 1)	P-value
Age (years)							
≤45	17	5	12		6	11	
>45	58	32	26	NS	28	30	NS
Gender							
Male	29	18	11		15	14	
Female	46	23	23	NS	20	26	NS
Tumor status							
pT1, pT2	16	9	7		6	10	
pT3, pT4	59	42	17	0.009	38	21	<0.001
Nodal status							
N0	40	22	18		23	17	
N1	35	25	10	0.035	5	30	0.011
Metastatic status							
M0	64	38	26		35	29	
M1	11	10	1	0.008	9	2	NS
Pathological subtype							
FTC	24	14	10	NS	12	9	NS
PTC	33	10	23	NS	10	19	NS
UTC	18	12	6	NS	2	9	NS

NS, not significant. FTC, follicular thyroid cancer; PTC, papillary thyroid cancer; UTC, undifferentiated thyroid cancer.
